# Modeling of Knudsen Layer Effects in the Micro-Scale Backward-Facing Step in the Slip Flow Regime

**DOI:** 10.3390/mi10020118

**Published:** 2019-02-12

**Authors:** Apurva Bhagat, Harshal Gijare, Nishanth Dongari

**Affiliations:** Department of Mechanical and Aerospace Engineering, Indian Institute of Technology, Hyderabad, Kandi, Medak 502285, India; me13m15p000001@iith.ac.in (A.B.); me13m15p000002@iith.ac.in (H.G.)

**Keywords:** rarefied gas flows, micro-scale flows, Knudsen layer, computational fluid dynamics (CFD), OpenFOAM, Micro-Electro-Mechanical Systems (MEMS), Nano-Electro-Mechanical Systems (NEMS), backward facing step

## Abstract

The effect of the Knudsen layer in the thermal micro-scale gas flows has been investigated. The effective mean free path model has been implemented in the open source computational fluid dynamics (CFD) code, to extend its applicability up to slip and early transition flow regime. The conventional Navier-Stokes constitutive relations and the first-order non-equilibrium boundary conditions are modified based on the effective mean free path, which depends on the distance from the solid surface. The predictive capability of the standard ‘Maxwell velocity slip—Smoluchwoski temperature jump’ and hybrid boundary conditions ‘Langmuir Maxwell velocity slip—Langmuir Smoluchwoski temperature jump’ in conjunction with the Knudsen layer formulation has been evaluated in the present work. Simulations are carried out over a nano-/micro-scale backward facing step geometry in which flow experiences adverse pressure gradient, separation and re-attachment. Results are validated against the direct simulation Monte Carlo (DSMC) data, and have shown significant improvement over the existing CFD solvers. Non-equilibrium effects on the velocity and temperature of gas on the surface of the backward facing step channel are studied by varying the flow Knudsen number, inlet flow temperature, and wall temperature. Results show that the modified solver with hybrid Langmuir based boundary conditions gives the best predictions when the Knudsen layer is incorporated, and the standard Maxwell-Smoluchowski can accurately capture momentum and the thermal Knudsen layer when the temperature of the wall is higher than the fluid flow.

## 1. Introduction

**Figure 1:**
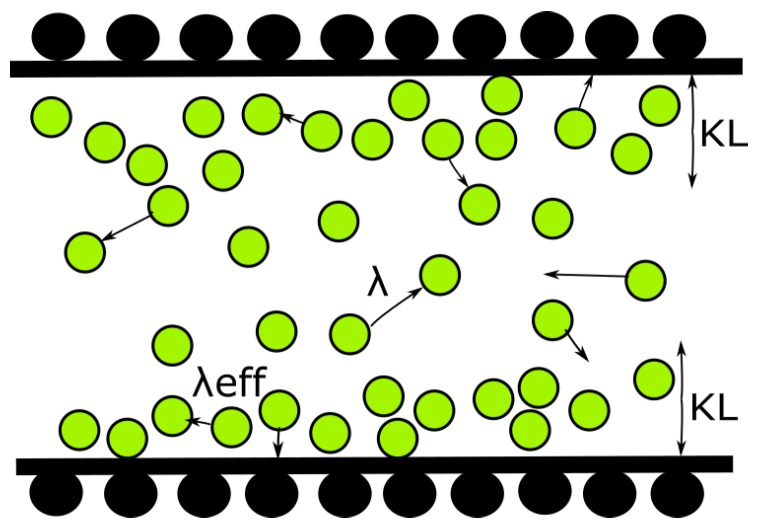
Schematic of inter-molecular and molecule-surface collisions leading to the formation of Knudsen layer (KL).

Conventional Navier-Stokes (NS) equations are based on the assumption that the mean free path (MFP) of the particle is much smaller than the characteristic length scale of the system. However, in a few engineering applications of interest, this continuum assumption deviates, if the flow is highly rarefied (e.g., vehicles operating at high altitude conditions), or length scale of the system is of the order of MFP of the gas (e.g., micro-scale gas flows). Flow through the nano-/micro-scale devices is dominated by non-equilibrium effects such as rarefaction and gas molecule-surface interactions. Knudsen layer (KL) is one such phenomenon, where a non-equilibrium region is formed near the solid surface in rarefied/micro-scale gas flows. Molecule-surface collisions are dominated by the presence of a solid surface reducing the mean time between collisions, i.e., unconfined MFP of the gas is effectively reduced in the presence of a solid surface [1]. Molecules collide with the wall more frequently than with other molecules, leading to the formation of the Knudsen layer as demonstrated in Figure 1. Linear constitutive relations for shear stress and heat flux are no longer valid in this region [2,3,4].

Behavior of the non-equilibrium gas flows and structure of KL have been extensively investigated by directly solving the Boltzmann equation [5], kinetic equations (e.g., the BGK (Bhatnagar, Gross and Krook) model, rigid-sphere model, the Williams model) [6,7,8,9] or alternative hydrodynamic models such as the Burnett equation, super-Burnett equations, Grad 13 moment equation and the regularized Grad moment equations [2,10,11,12,13]. However, obtaining solutions using these models is computationally challenging due to the complicated structure of molecular collisions term, lack of well-posed boundary conditions and inherent instability. The direct simulation Monte Carlo (DSMC) technique [14,15] is one of the most accepted and reliable methods for solving gas flows in the non-equilibrium region. Collisions of some representative particles with each other and wall boundaries, are handled in a stochastic manner [16]. As a result, computational cost becomes pretty intensive in case of micro-scale flows due to high density and low flow velocity. A few researchers [17,18,19] have applied DSMC method to analyze gas flow through micro-channel, and statistical scatter have been a critical issue. A huge sample size is required to reduce the statistical scatter, which makes the DSMC simulation tedious and time-consuming. These difficulties can be overcome if NS equations are extended with higher-order constitutive relations and boundary conditions so that they can accurately capture the Knudsen layer and non-linear flow physics of micro-scale gas flows.

Few researchers have attempted to include non-equilibrium effects in NS framework from different viewpoints. Myong [13] has derived the second-order macroscopic constitutive equation from the kinetic Boltzmann equation and obtained analytical solutions to the KL in Couette flow within continuum frame-work. Li et al. [20] have proposed an effective viscosity model to account for the wall effect in the wall adjacent layer. Lockerby et al. [21] have introduced the concept of wall function into a scaled stress-strain rate relation by fitting the velocity profile obtained from the linearized Boltzmann equation. This idea has been further extended to obtain Kn dependent functions [22], power-law scaling of constitutive relations [23] and discontinues correction function for near wall and far wall region [24,25]. The key disadvantage of these models is that they usually contain some empirical parameters which are specific for the geometry and the flow conditions, and it is not very convenient to extract them for various practical applications. Unlike these models, Guo et al. [26] developed a model based on the effective mean free path in which the wall bounding effect is considered with an assumption that MFP follows an exponential probability distribution. On the other hand, Dongari et al. [27] have hypothesized that the MFP of molecules follow a power-law based distribution, which is also valid in thermodynamic non-equilibrium.

Although several attempts have been made to improve constitutive relations, not much attention is given to the wall boundary conditions. Most previous studies are based on the classical velocity slip boundary conditions, as Lockerby et al. [21] and Dongari et al. [28] have used the first order velocity slip boundary condition by replacing MFP with effective MFP. Generalized second order slip boundary condition for velocity has been used by a few researchers [23,26,29]. Also, studies have been limited to low-speed isothermal gas flows over simple geometries like planar surface and cylinder. The temperature jump boundary condition with KL effects within NS framework, in thermal rarefied gas flows, has been overlooked in the literature to the best of authors knowledge. Present work aims to bridge the gap in the literature and different non-equilibrium boundary conditions, for both velocity and temperature, have been extended using effective MFP model proposed by Dongari et al. [27,28]. This model is rigorously validated against molecular dynamics (MD), DSMC, and experimental data, and also compared with other theoretical models [30,31,32,33]. The backward-facing step geometry is chosen in this manuscript as the flow experiences adverse pressure gradient and the separation.

In the present work, the effective MFP model [27,28] has been implemented in NS frame-work in open source CFD tool OpenFOAM. The mean free path is modified based on local flow density, and linear constitutive relations for shear stress and heat flux are modified to account for the effect of KL. In addition to this, first-order boundary conditions, (i) Maxwell velocity slip [34], (ii) Smoluchwoski temperature jump [35], as well as (iii) Langmuir Maxwell [36] and (iv) Langmuir Smoluchwoski [36] are modified with the effective mean free path. The simulations are carried out over a 2D backward-facing step nano- and micro-channel in the slip and early transition flow regime (0.01 < Kn < 0.1, Kn is the non-dimensional Knudsen number defined as λ/L, to indicate the degree of rarefaction, and *L* is the length-scale of the system). The novel contribution of the present work is that NS equations, combined with KL based constitutive relations and boundary conditions, are investigated for the flows with separation and reattachment. Results are compared with DSMC data [37], and validity of the proposed method is investigated. Effect of change in Knudsen number, inlet flow and wall temperature on the flow properties such as velocity slip and temperature jump is studied.

## 2. Computational Methodology

OpenFOAM (Open Field Operation and Manipulation, CFD Direct Ltd, UK) is a popular open source, parallel friendly *CFD* software, which is based on C++ library tools and a collection of various applications (created using these libraries). Implementation of tensor fields, partial differential equations, boundary conditions, etc. can be handled using these libraries [38,39].

The *rhoCentralFoam* solver is used as a base solver in the present study. It is a density-based compressible flow solver based on the central-upwind schemes of Kurganov and Tadmor [40,41]. Calculation of transport properties, formulation of KL within NS equations, governing equations with non-linear constitutive relations, and non-equilibrium various boundary conditions are explained in the Section 2.1, Section 2.2, Section 2.3 and Section 2.4.

### 2.1. Transport Properties

Transport coefficients are obtained using kinetic theory treatment [4,42,43], and the dynamic viscosity is calculated as:

(1) $$\mu =2.6693\times 10^{-5}\frac{\sqrt{MT}}{d^{2}F(k_{B}T/\epsilon )},$$

where *M* is the molecular weight, *T* is the temperature and *d* is the characteristic molecular diameter. $F(k_{B}T/\epsilon )$ is the function of $k_{B}T/\epsilon )$, which gives the variation of the effective collision diameter as a function of temperature (values are obtained from Bird et al. [14]), where ϵ is a characteristic energy of interaction between the molecules and $k_{B}$ is the Boltzmann constant. Values of *d* and $\epsilon /k_{B}$ for different gases are associated with the Lennard-Jones potential, and are tabulated by Anderson et al. [44].

Thermal conductivity is calculated by Eucken’s relation [45]:

(2) $$\kappa =\mu \left(C_{p}+\frac{5}{4}R\right),$$

where $C_{p}$ is the specific heat capacity at constant pressure and *R* is the specific gas constant.

### 2.2. Knudsen Layer Formulation

Using kinetic theory of gases [42], Maxwellian mean free path of a gas can be expressed as,

(3) $$\lambda =\frac{\mu }{\rho }\sqrt{\frac{\pi }{2RT}},$$

where μ is obtained from Equation (1), and ρ is the gas density.

The geometry dependent effective MFP model proposed by Dongari et al. [27,28,46,47,48] is defined as,

(4) $$\lambda_{eff}=\lambda \beta ,$$

where β is the normalized MFP which is function of local MFP and normal distance from the solid surface ($\hat{y}$) defined as,

(5) $$\beta =1-\frac{1}{96}\left[{\left(1+\frac{\hat{y}}{\lambda }\right)}^{1-n}+2\sum_{j=1}^{7}{\left(1+\frac{\hat{y}}{\lambda \cos(j\pi /16)}\right)}^{1-n}+4\sum_{j=1}^{8}{\left(1+\frac{\hat{y}}{\lambda \cos((2j-1)\pi /32)}\right)}^{1-n}\right]$$

where exponent n=3. This function is based on the assumption that molecules follow a non-Brownian motion when flow is confined by a solid surface. Detailed mathematical derivation and formulation of β for planar and cylindrical surfaces can be obtained in references [28,47] (refer to Equation (12) in [28] for planar geometry and Equation (18) in [47] for non-planar geometry).

Using Equations (3) and (4), effective viscosity is calculated as:

(6) $$\mu_{eff}=\mu \beta .$$

MFP for thermal cases (i.e., if temperature gradient exists in the flow) can be expressed as $\lambda_{T}=1.922\lambda$ [5] for hard sphere molecules. It has been stated by Sone et al. [5,49] on the basis of solution of linearized Boltzmann equation for hard sphere molecular model. Therefore, effective MFP expression for thermal cases becomes:

(7) $$\lambda_{eff(T)}=\lambda_{T}\beta_{T},$$

where $\beta_{T}$ is the normalized MFP for thermal cases [28].

Using Equations (2), (3) and (7), effective thermal conductivity is calculated as:

(8) $$\kappa_{eff}=\kappa \beta_{T}.$$

One should note that the transport properties μ and κ of the fluid are initially calculated from the kinetic theory based transport model described in the Section 2.1. Their effective values, i.e., $\mu_{eff}$ and $\kappa_{eff}$ are obtained to achieve the non-linear form of constitutive relations, which account for the non-equilibrium effect of KL.

### 2.3. Governing Equations

The *rhoCentralFoam* solver computes the following governing equations, namely conservation of total mass, momentum and energy [50]:

(9) $$\frac{\partial \rho }{\partial t}+\nabla \cdot \left[\rho u\right]=0,$$

(10) $$\frac{\partial (\rho u)}{\partial t}+\nabla \cdot \left[u\left(\rho u\right)\right]+\nabla p+\nabla \cdot \Pi =0,$$

(11) $$\frac{\partial (\rho E)}{\partial t}+\nabla \cdot \left[u\left(\rho E\right)\right]+\nabla \cdot \left[up\right]+\nabla \cdot \left[\Pi \cdot u\right]+\nabla \cdot j=0,$$

where u is the velocity of the flow, *p* is pressure, $E=e+\frac{{\left|u\right|}^{2}}{2}$ is the total energy, *e* is specific internal energy, and Π is the shear stress tensor calculated as:

(12) $$\Pi =\mu_{eff}\left(\nabla u+{\left(\nabla u\right)}^{T}-\frac{2}{3}Itr\left(\nabla \cdot u\right)\right),$$

where $\mu_{eff}$ is the effective shear viscosity of the fluid, which accommodates non-linearity due to KL effects (see Equation (6)), and, **I** and *tr* denotes, identity matrix and trace, respectively. The heat flux due to conduction of energy (j) by temperature gradients (Fourier’s law) is defined as:

(13) $$j=-\kappa_{eff}\nabla T,$$

where $\kappa_{eff}$ is the effective thermal conductivity of the fluid based on effective thermal MFP (see Equation (8)). And temperature is calculated iteratively from the total energy as:

(14) $$T=\frac{1}{C_{v}\left(T\right)}\left(E\left(T\right)-\frac{{\left|u\right|}^{2}}{2}\right),$$

where $C_{v}\left(T\right)$ is the specific heat at constant volume as a function of temperature.

Perfect gas equation is solved to update the pressure as:

(15) p=ρRT.

### 2.4. Boundary Conditions

The first-order Maxwell velocity slip boundary condition [34], is modified to take into account the KL correction [47] as follows:

(16) $$u=u_{w}-\left(\frac{2-\sigma_{v}}{\sigma_{v}}\right)\lambda_{eff}\nabla_{n}\left(S\cdot u\right)-\left(\frac{2-\sigma_{v}}{\sigma_{v}}\right)\frac{\lambda_{eff}}{\mu_{eff}}S\cdot \left(n\cdot \Pi_{\mathrm{mc}}\right)-\frac{3}{4}\frac{\mu_{eff}}{\rho }\frac{S\cdot \nabla T}{T},$$

where $u_{w}$ is the reference wall velocity, $\sigma_{v}$ is tangential momentum accommodation coefficient, subscript *n* denotes normal direction to the surface, the tensor S = **I** − **nn** removes normal components of non-scalar field, and $\Pi_{\mathrm{mc}}=\Pi -\mu_{eff}\nabla u$ is obtained from Equation (12). Here, third term on the RHS of Equation (16) accounts for the curvature effect and fourth term considers the thermal creep.

Smoluchowski temperature jump [35] is modified as follows:

(17) $$T=T_{w}-\frac{2-\sigma_{T}}{\sigma_{T}}\frac{2\gamma }{\gamma +1}\frac{\lambda_{eff(T)}}{Pr}\nabla_{n}T,$$

where $T_{w}$ is the reference wall temperature, Pr is Prandtl number, $\sigma_{T}$ is thermal accommodation coefficient and γ is specific heat ratio.

In addition to above widely used boundary conditions, following hybrid boundary conditions, which consider the effect of adsorption of molecules on the surface, are also evaluated in the present work. These boundary conditions are developed by Le et al. [36], and have proven to give good results for rarefied hypersonic flow cases, and low-speed rarefied micro-scale gas flows [37]. These boundary conditions are based on the concept that the molecules are adsorbed by the solid surface, as a function of pressure at constant temperature. If molecules are adsorbed by the fraction α, they do not contribute to the fluid shear stress and conduction of heat due to receding molecules (1 −α). This fraction of coverage α is computed by the Langmuir adsorption isotherm [51,52] for mono-atomic gases,

(18) $$\alpha =\frac{\zeta p}{1+\zeta p},$$

and for diatomic gases,

(19) $$\alpha =\frac{\sqrt{\zeta p}}{1+\sqrt{\zeta p}},$$

where ζ is an equilibrium constant related to surface temperature, which is represented as,

(20) $$\zeta =\frac{A_{m}\lambda_{eff}}{R_{u}T_{w}}exp\left(\frac{D_{e}}{R_{u}T_{w}}\right),$$

where $A_{m}$ is approximately calculated as $N_{A}\pi d^{2}/4$ for gases [52,53], $N_{A}$ is Avogadros’s number, $D_{e}$ = 5255 (J/mol) is the heat of adsorption for argon and nitrogen given in literature [52,53], and $R_{u}$ is the universal gas constant.

Langmuir-Maxwell slip velocity [36] boundary condition is modified as,

(21) $$u=u_{w}-\left(\frac{1}{1-\alpha }\right)\lambda_{eff}\nabla_{n}\left(S\cdot u\right)-\left(\frac{1}{1-\alpha }\right)\frac{\lambda_{eff}}{\mu_{eff}}S\cdot \left(n\cdot \Pi_{\mathrm{mc}}\right)-\frac{3}{4}\frac{\mu_{eff}}{\rho }\frac{S\cdot \nabla T}{T},$$

and Langmuir-Smoluchwoski temperature jump [36] boundary condition is modified as,

(22) $$T=T_{w}-\frac{1}{1-\alpha }\frac{2\gamma }{\gamma +1}\frac{\lambda_{eff(T)}}{Pr}\nabla_{n}T.$$

These boundary conditions consider the effect of KL as well as adsorption on the wall.

It should be noted that all equations stated above reduce to their conventional form when $\beta =\beta_{T}=1$. All simulations are carried out using the conventional *rhoCentralFoam* solver without the effect of KL initially. Local MFP (λ) in Equation (3) and the geometry-dependent effective MFP ($\lambda_{eff}$) in Equation (4) are updated using the post-processing utility developed by authors within OpenFOAM framework and simulations are carried out again. Results obtained using conventional NS equations, using linear constitutive relations, along with Maxwell velocity slip and Smoluchwoski temperature jump (MS) are referred as *“NS-MS”*, whereas, Langmuir-Maxwell velocity slip and Langmuir-Smoluchwoski temperature jump (LMS) boundary conditions are referred as *“NS-LMS”* throughout the manuscript. Current results, which are referred as *“NS-MS-withKL”* and *“NS-LMS-withKL”* are obtained using the modified constitutive relations and respective boundary conditions with effective MFP (β and $\beta_{T}$). Flow is modeled using a single gas species in chemical equilibrium in the present study.

## 3. Results and Discussion

**Figure 2:**
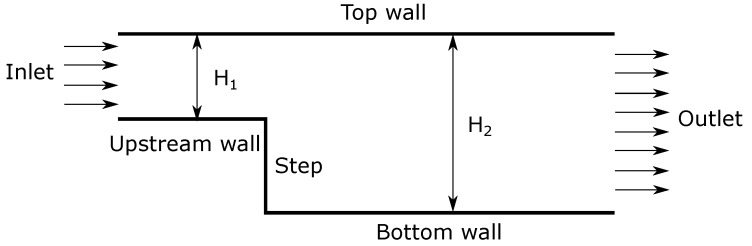
Schematic of backward-facing step.

A schematic of the backward-facing step is illustrated in Figure 2. Dirichlet boundary condition is imposed for pressure at inlet and outlet, whereas zero-gradient boundary condition is used for velocity (extrapolated from the interior solution), as it is a pressure driven flow. The temperature of flow is specified at the inlet boundary and zero-gradient at the outlet. Various non-equilibrium surface boundary conditions (described in Section 2.4) have been applied at the top wall, upstream wall, step, and bottom wall. Dimensions of the nano-/micro step channel vary depending on the Knudsen number and are given in Table 1. The authors have compared the results with the DSMC data obtained by Mahadavi et al. [37]. Specified inlet and outlet pressure boundary conditions have been implemented in DSMC simulations, through correcting density and velocity implicitly from the characteristics theory [54,55]. A fully diffuse reflection wall patch (perfect exchange of momentum and energy), which corresponds to $\sigma_{v}=\sigma_{T}=1$ in CFD simulations, has been used for all simulations.

**Figure 3:**
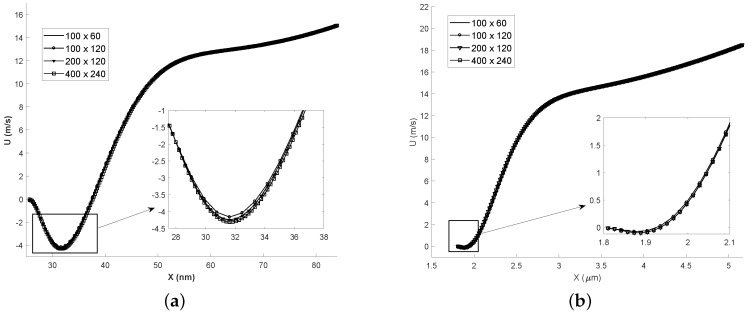
Grid independence study by analysing the slip velocity distribution on the bottom wall. Legends show the number of cells in *x* direction (along the length of channel) × *y* direction (along the height of channel). (**a**) Kn = 0.01; (**b**) Kn = 0.05.

A grid is created using the ‘blockMesh’ utility in OpenFOAM. A grid independence study has been carried out by gradually increasing cells in *x* and *y*-direction as shown Figure 3. Slip velocity on the bottom wall is plotted for nano-channel (refer Figure 3a) and micro-channel (refer Figure 3b). Results obtained are independent of the grid resolution. Final grid size have 200 cells in *x*-direction (minimum cell size δx = 0.427 nm) and 120 cells in y-direction (minimum cell size δy = 0.142 nm) for nano-channel, and micro-channel grid has 300 cells in *x*-direction (δ*x* = 0.0187 μm) and 60 cells in *y*-direction (δ*y* = 0.0166 μm).

### 3.1. Effect of Change in Knudsen Number

In this section, simulations are carried out for various Knudsen numbers in slip (Kn = 0.01, 0.05), and early transition (Kn = 0.1) flow regime. Flow parameters for all 3 cases are given in Table 2. The temperature of the inlet flow and wall is same for Kn = 0.01 case, and minimal difference of 30 K for other 2 cases. The nano-step channel is used for Kn = 0.01 case, whereas a micro-step channel is used to simulate high Kn cases [37]. Although the height of backward-facing step is less for Kn = 0.01 case, inlet pressure is very high compared to higher Kn cases. Slip velocity distribution obtained using different solvers on the bottom wall of the backward-facing step is plotted. Gradient-based local Kn ($Kn_{gll}=\frac{1}{Q}\frac{dQ}{dl}$) is plotted on the secondary *y*-axis for all plots. Here, $Kn_{gll}$ is calculated based on velocity gradients and *Q* in the denominator is taken as maximum of ($u,\sqrt{\gamma RT}$).

**Figure 4:**
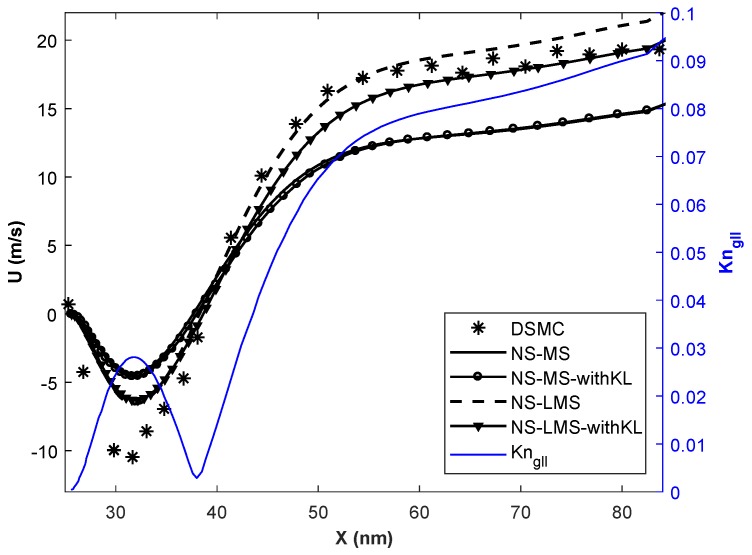
Velocity slip distribution on the bottom wall at Kn = 0.01.

Figure 4 shows the slip velocity distribution for Kn = 0.01 case i.e., slip flow regime. Flow accelerates through the nano-step channel along its length and undergoes Prandtl-Meyer expansion at the upstream wall-step corner. Flow is separated from the wall, and a wake is formed immediately after the step. Negative slip velocity components in Figure 4 for x < 38 nm, indicates the reverse flow and the adverse flow gradient. Flow is reattached to the wall at x = 38 nm, and slip velocity increases along the streamline. It is observed that in the separation region, solvers using LMS boundary conditions give better results than usual MS boundary conditions when compared with DSMC data. Also, the introduction of KL effects in NS-MS solver does not change results, as they exactly overlap with each other. Their deviation w.r.t. DSMC increases as flow becomes more rarefied towards the outlet, maximum deviation being 26.66%. On the other hand, results are considerably improved when KL effects are incorporated in NS-LMS solver, and they are in excellent agreement towards the outlet. Flow is more rarefied near outlet, as $Kn_{gll}$ is higher, which leads to the growth of thickness of KL. However, with and without KL results are similar in the separation zone (x < 38 nm), as local Kn < 0.03, and KL effects are minimal in this region.

**Figure 5:**
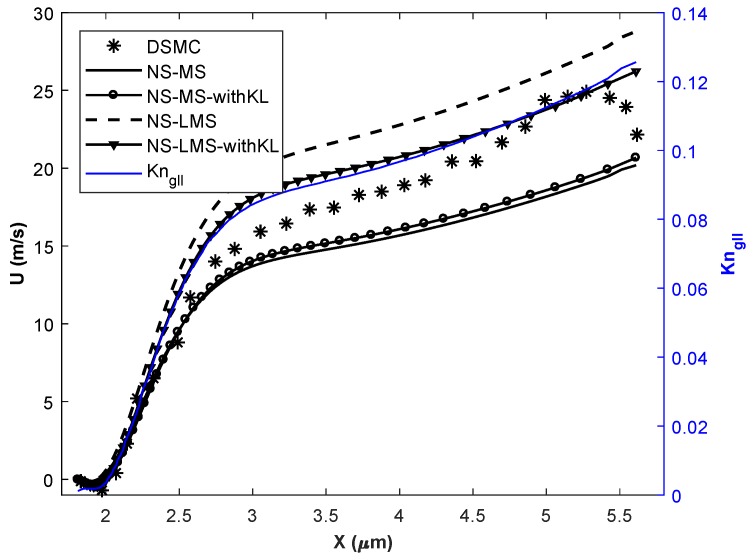
Velocity slip distribution on the bottom wall at Kn = 0.05.

**Figure 6:**
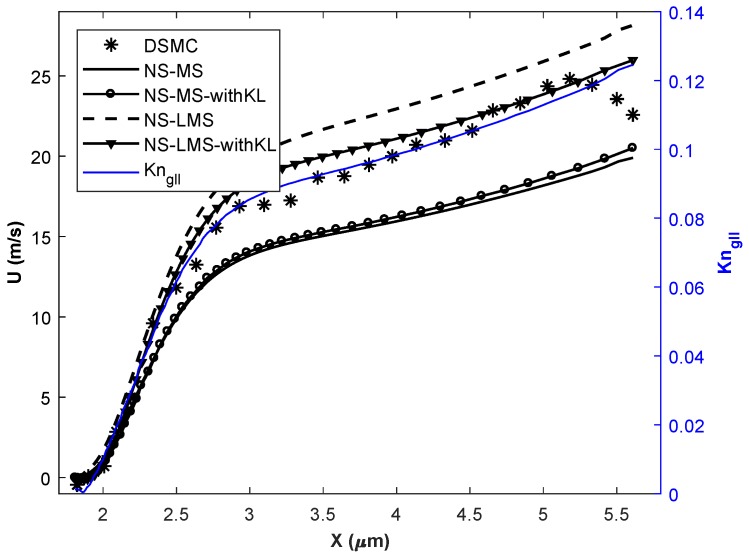
Velocity slip distribution on the bottom wall at Kn = 0.1.

**Figure 7:**
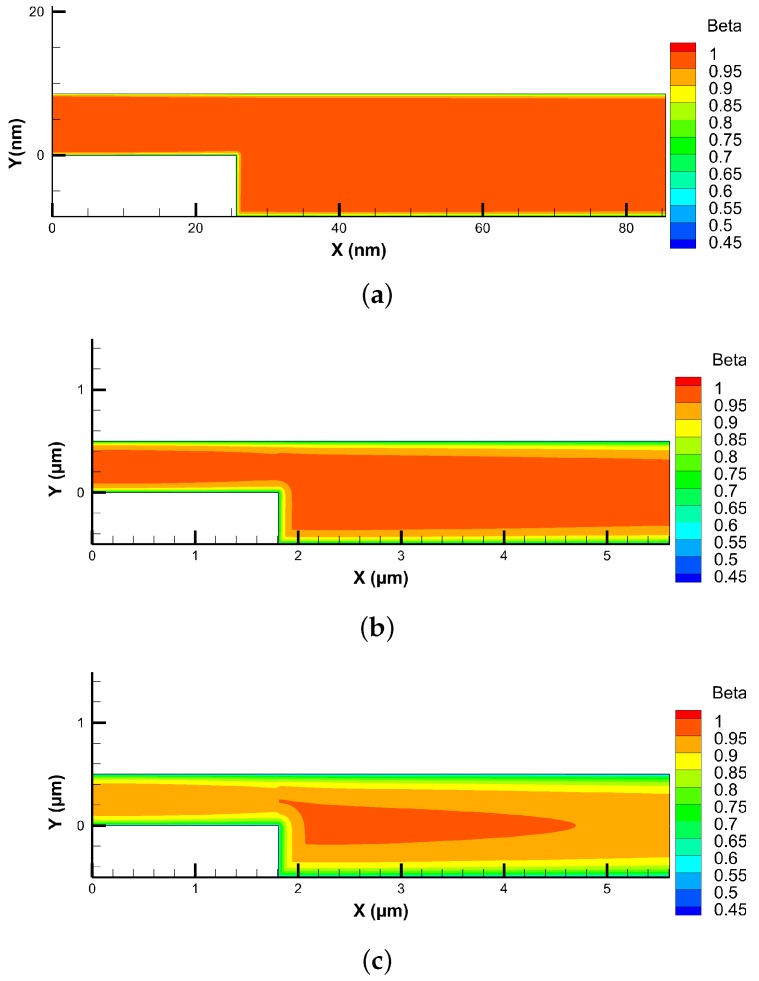
Knudsen layer formation in terms of the normalized MFP (β) contours for various Knudsen numbers (obtained using Equation (5)). (**a**) Kn = 0.01; (**b**) Kn = 0.05; (**c**) Kn = 0.1.

Figure 5 and Figure 6 demonstrate the slip velocity distribution on the bottom wall for Kn = 0.05 and 0.1 cases, respectively. As the flow enters the early transition regime, the phenomena of flow separation, recirculation, and re-attachment disappear. This is because slip velocity on the wall becomes comparable to the flow velocity. The Knudsen layer thins the shear layer, and fluid follows wall direction without separating even at the sudden step corner. It can be observed that NS-MS solver under-predicts, whereas NS-LMS solver over-predicts the slip velocity when compared to DSMC, throughout the length of the bottom wall. After the incorporation of KL effects, slip velocity values predicted by NS-MS solver, move closer to DSMC data, but the improvement is not much significant (∼5.88%), and maximum deviation with DSMC is ∼ 31.57%. On the other hand, results are greatly improved for NS-LMS-KL solver, i.e., 10% improvement over NS-LMS solver and deviations within 10% with DSMC data. It is noticed that the introduction of KL is more effective when Kn${}_{gll}$ > 0.05, as the growth of KL thickness increases with increase in Kn. Visualization of KL thickness for various Kn cases is demonstrated in Figure 7, using contours of normalized MFP (β). Thickness of KL is minimal for the Kn = 0.01 case, whereas it almost covers the entire flow domain for Kn = 0.1 case.

### 3.2. Effect of Change in Inlet Temperature

In this section, the temperature of the flow at the inlet is varied to investigate the effect of KL on temperature jump on the bottom wall. Simulations are carried out for Kn = 0.01 case with T${}_{in}$ = 500 K and T${}_{in}$ = 700 K.

**Figure 8:**
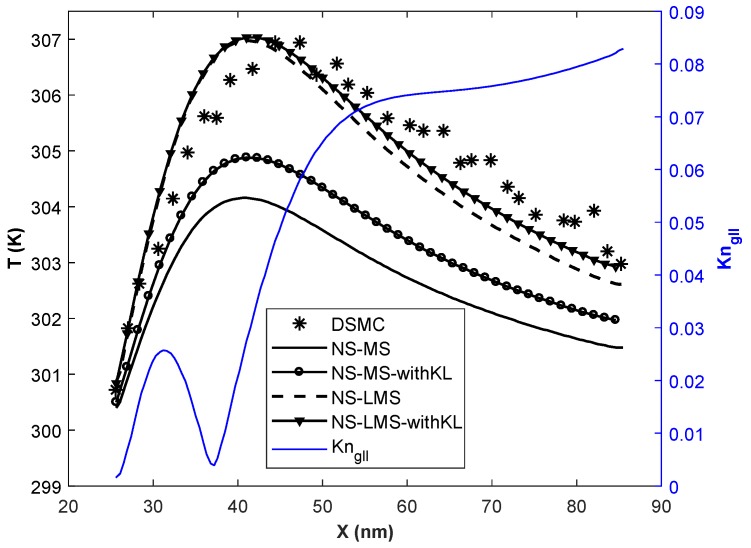
Temperature distribution of the fluid on the bottom wall at Kn = 0.01, T${}_{in}$ = 500 K.

**Figure 9:**
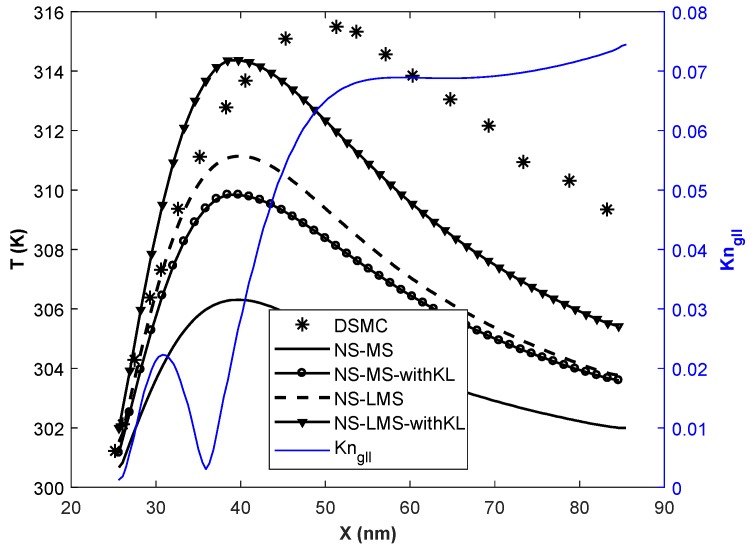
Temperature distribution of the fluid on the bottom wall at Kn = 0.01, T${}_{in}$ = 700 K.

Figure 8 and Figure 9 demonstrate the temperature of the fluid on the bottom wall obtained using different solvers. Local Kn based on velocity gradients is plotted on the secondary y-axis. It is observed that NS-LMS solver results are closer to DSMC data when compared to NS-MS solver. This is due to the fact that surface boundary conditions in the DSMC method are based on gas-surface interactions, and particles are adsorbed on the surface and re-emitted. LMS boundary conditions account for the effect of molecules adsorption on the surface and are able to predict better surface properties than MS boundary conditions.

However, the introduction of KL effects has noticeably improved predictions for both the solvers, NS-MS and NS-LMS. The main reason behind this is that the thermal KL is formed near the wall, whose thickness is more than the momentum KL. It not only modifies the constitutive relation for the heat flux but also considers the thermal MFP in the calculation of surface temperature jump. The introduction of KL effects has improved NS-MS solver results even in the separation zone, with maximum relative improvement of 30% for T${}_{in}$ = 500 K case, and 41.05% for T${}_{in}$ = 700 K case w.r.t. DSMC results. NS-LMS solver with and without KL accurately captures the peak temperature value for T${}_{in}$ = 500 K case. Location of peak temperature for DSMC is slightly downstream of NS solutions, as flow gradients in DSMC are diffuse. Relative improvement of around 81% is observed for T${}_{in}$ = 700 K case solver due to the addition of KL effects over NS-LMS solver.

### 3.3. Effect of Change in Wall Temperature

In this section, the temperature of the bottom wall and step has been varied for Kn = 0.01 case. Inlet flow and other walls have the temperature of 300 K. Simulations are carried out by setting temperature of both the bottom wall and step to 500 K and 700 K.

**Figure 10:**
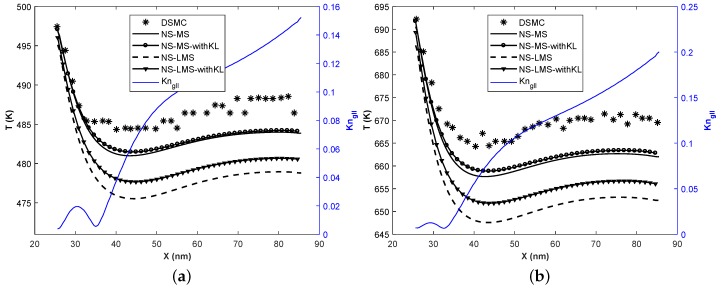
Temperature distribution of the fluid on the bottom wall at Kn = 0.01. (**a**) T${}_{w}$ = 500 K ; (**b**) T${}_{w}$ = 700 K.

Figure 10 shows the temperature distribution of fluid on the bottom wall for T${}_{w}$ = 500 K (Figure 10a) and T${}_{w}$ = 700 K (Figure 10b). As wall temperature is higher than the flow temperature, heat is transferred from the wall to the fluid flow, and temperature abruptly drops near the step - bottom wall region where a separation zone is formed.

It is interesting to note that for this particular case, NS-MS solvers predict better surface temperature than NS-LMS solver, unlike previous cases. This can be attributed to the fact that reference temperature T${}_{w}$ is high, which affects the calculation of α and ζ parameters in Equations (19) and (20), respectively. In LMS boundary conditions, approaching stream of molecules has lower temperature than the surface, and high temperature surface adsorbs molecules by fraction of α. These molecules are re-emitted with T = T${}_{w}$, which could be the reason that temperature is under-predicted by NS-LMS solvers. Therefore, LMS boundary conditions are not suitable when temperature gradients are negative or not uniform. Though NS-LMS solver has deviations w.r.t DSMC data, KL effects have led to the relative improvement of around 24% for T${}_{w}$ = 500 K case and 29% for T${}_{w}$ = 700 K case. Results are minutely improved for NS-MS solvers with the addition of KL effects. Peak temperature values are accurately captured by NS-MS-withKL solver.

**Figure 11:**
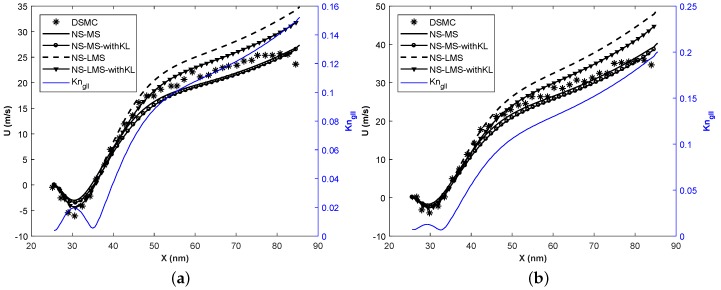
Velocity slip distribution on the bottom wall at Kn = 0.01. (**a**) T${}_{w}$ = 500 K ; (**b**) T${}_{w}$ = 700 K.

Figure 11 demonstrates the comparison of slip velocity for various solvers for T${}_{w}$ = 500 K (Figure 11a) and T${}_{w}$ = 700 K (Figure 11b) cases. It is observed that NS-LMS solver gives better predictions in separated region, however, it overpredicts the slip velocity after reattachment i.e., x > 35 nm for T${}_{w}$ = 500 K and x > 32 nm for T${}_{w}$ = 700 K case. Slip velocity moves closer to DSMC when KL effect is taken into account in NS-LMS solver. As described earlier, KL promotes shear thinning phenomenon, which leads to less slip velocity on the wall, and higher normal gradients of velocity on the wall. There is a minor discrepancy between DSMC and NS-MS, NS-MS-withKL solvers for T${}_{w}$ = 500 K case, whereas there is a good agreement for T${}_{w}$ = 700 K case.

## 4. Conclusions

The effect of the Knudsen layer on the surface flow properties of a nano- and micro-scale backward facing step has been investigated in the slip and the early transition flow regime. The effective mean free path model has been implemented within the Navier-Stokes framework, and the constitutive relations for shear stress and heat flux are modified. First order non-equilibrium boundary conditions, i.e., Maxwell velocity slip and Smoluchowski temperature jump, as well as hybrid boundary conditions based on Langmuir adsorption isotherm are effectively modified to incorporate the non-equilibrium effects associated with KL.

The NS-LMS solver has proven to give better predictions in the separation zone than NS-MS solver when compared with the benchmark DSMC results. The velocity slip and temperature jump results are significantly improved for the NS-LMS method when KL effects are incorporated, implying that non-linear effects due to momentum and thermal KL are captured by the proposed method. On the other hand, for the case of negative temperature gradient near the wall, the NS-MS solver have accurately predicted the slip velocity and temperature jump and has good agreement with DSMC data, and NS-LMS method have higher deviations with DSMC.

The present results have demonstrated the potential of the effective MFP based approach in the modeling of rarefied nano- and micro-scale gas flows. Although non-linear effects associated with momentum and thermal KL are captured up to some extent, no strong conclusions can be drawn. In the future, the detailed analysis should be carried out over a wide range of Knudsen numbers, and arbitrary geometries subjected to a range of complex flow conditions.

## Figures and Tables

**Table 1:** Dimensions of nano- and micro-step channel.

Dimensions of	Nano-Step Channel	Micro-Step Channel
Top wall	85.47 nm	5.61 μm
Upstream wall	25.641 nm	1.81 μm
Bottom wall	59.829 nm	3.8 μm
H${}_{1}$	17.095 nm	1 μm
H${}_{2}$	8.547 nm	0.5 μm
Step	8.547 nm	0.5 μm

**Table 2:** Flow parameters for various Kn number cases.

Kn (Based on H${}_{2}$)	0.01	0.05	0.1
P${}_{in}$ (MPa)	31.077	0.150735	0.075397
T${}_{in}$(K)	300	330	330
P${}_{in}$/P${}_{out}$	2	2.32	2.32
T${}_{w}$(K)	300	300	300
$\sigma_{v},\sigma_{T}$	1	1	1
Geometry	Nano-step	Micro-step	Micro-step
Gas	N${}_{2}$	N${}_{2}$	N${}_{2}$
